# Novel GII.12 Norovirus Strain, United States, 2009–2010

**DOI:** 10.3201/eid1708.110025

**Published:** 2011-08

**Authors:** Everardo Vega, Jan Vinjé

**Affiliations:** Author affiliation: Centers for Disease Control and Prevention, Atlanta, Georgia, USA

**Keywords:** viruses, enteric infections, gastroenteritis, foodborne diseases, CalciNet, GII.12, norovirus, outbreaks, novel strain, recombinant virus, United States, dispatch

## Abstract

In October 2009, a novel GII.12 norovirus strain emerged in the United States and caused 16% of all reported norovirus outbreaks during the winter season. Sequence analysis demonstrated a recombinant virus with a P2 region that was largely conserved compared with previously sequenced GII.12 strains.

Noroviruses are the leading cause of viral gastroenteritis outbreaks in the United States (*1*). Over the past decade most norovirus outbreaks have been caused by genogroup (G) II.4 noroviruses, while each of the other genotypes did not cause >7% of the outbreaks (*2**,**3*). However, previous studies have suggested that non-GII.4 noroviruses have been predominant in the past (*3**,**4*). For example, analysis of archived samples from 1974 through 1991 has shown that the frequency of GII.3 was 48% compared with 16% for GII.4 and 14% for GII.7 strains (*3*). Therefore, it is essential to study sudden increases of non-GII.4 strains to determine possible signatures that could be associated with increased transmissibility or population susceptibility. In this article, we describe the emergence of a novel GII.12 strain in the United States in the winter of 2009–10 that was associated with a large number of the norovirus outbreaks.

## The Study

**Figure 1:**
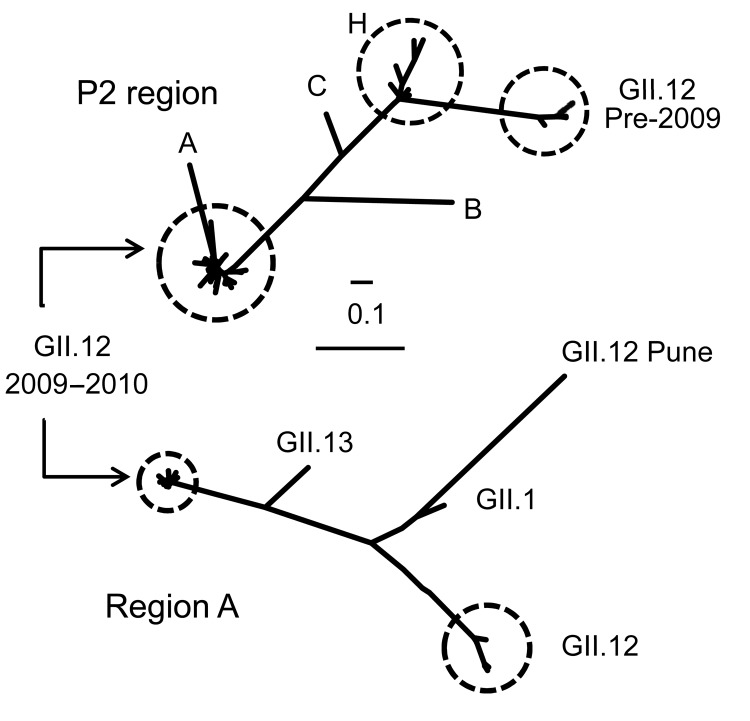
Phylogenetic trees of the P2 region in open reading frame (ORF) 2 and region A in ORF1 of noroviruses, United States. The P2 region and region A phylogenetic trees include GII.12 sequences from strains submitted to GenBank and GII.12 sequences reported in this study. In addition, region A analysis includes GII.12 Pune (GenBank accession no. EU921353), GII.1 (accession no. U07611), and GII.13 (accession no. DQ379714) sequences. Branch identifiers for the P2 region are as follows, with GenBank accession numbers in parentheses: A) Pune (EU921353); B) Pirna (AF427119); C) Wortley (AJ277618); H) Akabane (EF547403), Hiroshima (AB044366), Chitta virus (AB032758), Honolulu (AF414420), Gifu’96 (AB045603), U1GII (AB067536), U1 (AB039775), and Schwerin (AF397905). The GII.12 phylogenetic tree of the P2 region and region A region include GII.12 sequences StGeorge (accession no. GQ845370), Shelby (accession no. HQ688986), and all GII.12 noroviruses from 2009 through June 2010 (not distinguishable from GII.12 2009–10 cluster). The P2 region analysis also includes the Velence strain (accession no. HQ115742). Scale bars indicate nucleotide substitutions per site.

**Figure A1:**
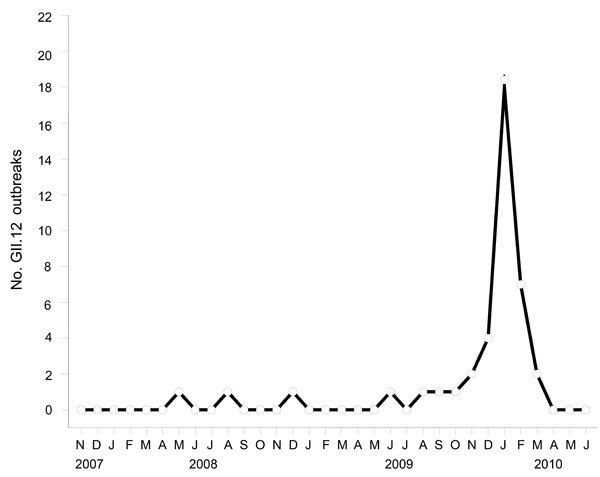
Number of GII.12 norovirus outbreaks reported to the Centers for Disease Control and Prevention November 2007–June 2010.

From October 2009 through June 2010, fecal specimens from patients affected by 194 outbreaks from 21 states were submitted to the Centers for Disease Control and Prevention (CDC, Atlanta, GA, USA); 39 (20%) of the viruses were typed as GII.12 by phylogenetic analysis by using region D sequences (*5*). During the same period, CaliciNet data confirmed an identical GII.12 strain that caused 67 (14%) of the 469 outbreaks reported by 12 states (*6*). To further study these new strains, we amplified the P2 region from 38 GII.12 outbreaks and 3 GII.12 strains reported to the Centers for Disease Control and Prevention from 2007 through March 2010 (Figure A1) using the SuperScript III One-Step RT-PCR System with Platinum Taq High Fidelity (Invitrogen, Carlsbad, CA, USA). The final reaction mix consisted of 400 nM of oligonucleotide primers EVP2GII12F, 5′-ATC TAA TGG YTC TGG TGA TGA TG-3′ and EVP2GII12R, 5′-YGC CAC ACC TCC TTT AAG AG-3′. The primers annealed at positions 1132 and 1891 of the GII.12 strain Honolulu (GenBank accession no. AF414420) to yield a product of 759 bp. Cycling conditions included reverse transcription for 30 min at 48°C; denaturation for 2 min at 94°C; followed by 40 cycles of 94°C for 15 s, 48°C for 30 s, 68°C for 1 min; and a final extension step of 68°C for 5 min. A 2.5-kb region, including the complete open reading frame (ORF) 2 and partial ORF3 genes, was amplified by using GII conserved primers RING2-PCR (5′-TGG GAG GGC GAT CGC AAT CT-3′) and PanGIIR1 (5′-GTC CAG GAG TCC AAA A-3′). The primers annealed at positions 535 and 2888 of the GII.12 strain Honolulu (AF414420) to yield a product of 2.3 kb. The GII.12 P2 sequences were similar to 2 GII.12 strains detected in sporadic cases in Australia (*7*) and Hungary in 2009 (Figure 1).

**Figure 2:**
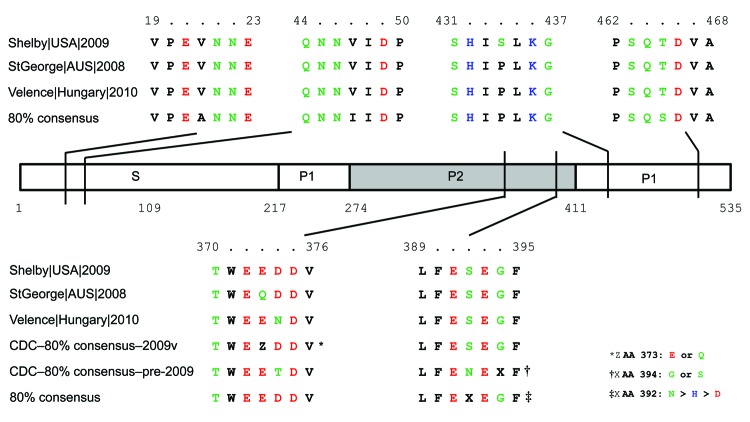
GII.12 norovirus viral protein (VP) 1 cartoon depicting amino acid similarities and locations between 2009–10 GII.12 strains and pre-2009 GII.12 strains. The S, P1, and P2 domains of VP1 are labeled accordingly. The VP1 amino acid numbering is based on the GII.12 prototype strain Wortley (GenBank accession no. AJ277618). Amino acid types are indicated by colors: green, polar; blue, basic; red, acidic; and black, hydrophobic. The 80% consensus sequence is based on a VP1 consensus sequence from the following GII.12 strains: GenBank accession nos. EU921353, AF427119, AJ277618, EF547403, AB044366, AB032758, AF414420, AB045603, AB067536, AB039775, and AF397905. Three recombinant 2009–2010 GII.12 strains—StGeorge (accession no. GQ845370), Velence (accession no. HQ115742), and Shelby (accession no. HQ688986)—were not included. The Centers for Disease Control and Prevention (CDC)–80% consensus–2009v and CDC–80% consensus–pre-2009 sequences are based on an 80% consensus P2 sequence from samples received from 2009–2010 (2009v) or from sample before 2009 (pre-2009), respectively.

Phylogenetic analysis of P2 sequences from all GII.12 strains indicated a temporal pattern, with the new strains clustering separately from GII.12 strains detected before 2009 (Figure A1). The new GII.12 strains clustered with GII.12 strains from Australia and Hungary in both the P2 and region A (*5*) (Figure 1). A single amino acid change in P2, at aa 392, occurred consistently in the new strains compared with archival GII.12 noroviruses (Figure 2). Additional amino acid substitutions were identified outside the P2 region at positions 22, 47, and 465 (Figure 2). Partial RNA-dependant RNA polymerase sequences confirmed that the new GII.12 strains were recombinant viruses, as reported previously (*7*) (Figure 1). Because different norovirus polymerases may have different nucleotide incorporation rates (*8*), and thus could play a role in enhanced replication efficiency of the new GII.12 strains, we amplified and analyzed a partial region of the polymerase gene but found no differences between the GII.12 strains pre- or post-2009.

## Conclusions

A novel GII.12 norovirus strain emerged in winter 2009–10 and caused 16% of the norovirus outbreaks in the United States. Sequence comparison with archival GII.12 strains demonstrated that even though there were clear and distinct nucleotide changes in the P2 region, there were few amino acid changes in the complete viral protein (VP) 1 (Figure 2). The increase in the number of GII.12 outbreaks (Figure A1) was confirmed by data from CaliciNet, which indicates that the increase was not caused by surveillance bias (*6*). Both P2 as well as polymerase sequence analysis demonstrated the emergence of a recombinant strain without novel amino acid substitutions in the P2 region, as has been reported for emerging GII.4 variants (*9*). All nucleotide substitutions throughout the P2 region of the new GII.12 strains, compared with pre-2009 GII.12 strains, were synonymous or unstable mutations (Figure 2).

Most norovirus evolution studies have focused on the VP1, more specifically the P2 region, where the antigenic and histo–blood group antigen attachment sites are located (*7**,**9**,**10*). The novel GII.12 virus had 1 unique amino acid substitution in the P2 domain but with similar biochemical properties, suggesting that the emergence of this virus may have been caused by virulence features coded by signatures outside of the P2 region or by changes in population susceptibility. In contrast to a recent report (*11*), our results suggest that the nonstructural proteins or regions outside of the P2 region may play a role in the evolution and virulence of norovirus strains. Substitutions in some key amino acids in the N-terminal region of the capsid have been speculated to be involved in the formation of secondary structures for efficient initiation of translation of VP1 (*12*).

The fact that 16% of all reported outbreaks were caused by a rare genotype highlights the importance of norovirus strain typing, which may provide insights into identifying which viral and/or host factors enable the emergence of novel norovirus strains. CaliciNet, which demonstrated its usefulness in this study, will be an important tool for monitoring changing trends and emergence of novel strains. However, until a cell culture system, small animal model, or infectious clone for human norovirus is available, the role of structural or nonstructural genes on norovirus pathogenicity and transmissibility will be difficult to assess.
